# Orthodontic Space Closure of a Missing Maxillary Lateral Incisor Followed by Canine Lateralization

**DOI:** 10.1155/2020/8820711

**Published:** 2020-11-14

**Authors:** Sanjay Prasad Gupta, Shristi Rauniyar

**Affiliations:** ^1^Orthodontics and Dentofacial Orthopedics Unit, Department of Dentistry, Tribhuvan University Dental Teaching Hospital, Institute of Medicine, Tribhuvan University, Kathmandu, Nepal; ^2^Dental Villa-Orthodontic Center & Speciality Dental Clinic, Kathmandu, Nepal

## Abstract

Maxillary lateral incisor agenesis is the most prevalent developmental dental anomaly. The management of missing lateral incisor was either closure using canine as substitution or creation of space orthodontically for prosthetic replacement. A careful diagnosis and treatment plan are deemed essential to address the patient's needs as the spacing is present in the esthetic region. Such problem is very challenging for orthodontists, prosthodontists, and general practitioners. This case report describes the orthodontic management of a 22-year-old adult female patient with missing upper left lateral incisor tooth and upper anterior spacing by closing the space with canine lateralization and reshaping to simulate it with the lateral incisor. However, some modifications in the treatment mechanics are crucial to achieve the optimal esthetic and to improve the occlusion. Space closure with canine lateralization option seems less invasive, treatment can be completed relatively in a short period of time, and its adaptation with the facial changes throughout life without having artificial prosthesis provided other factors favoring for this option.

## 1. Introduction

Variations in the form of maxillary lateral incisors are more than any other tooth in the mouth except the third molars. Missing maxillary lateral incisor (agenesis) is the most common developmental anomaly [1, 2]. The prevalence of agenesis of maxillary lateral incisor among Nepalese orthodontic patients is 5.82% [3].

Studies revealed that its prevalence rate was more among Asians and common in females [4–6]. There may be multiple reasons for the agenesis of maxillary lateral incisor like trauma, infection, medication, mutation of genes (MSX and PAX9), and some syndromes including ectodermal dysplasia, Down syndrome, and cleft lip and palate [7].

Patients with missing teeth may suffer from a reduced chewing ability, inarticulate pronunciation, and an unfavorable esthetic appearance that ultimately affects their communication behavior, self-esteem, and professional performance [8].

The management of missing teeth, especially lateral incisors, was either closure using canine as substitution (canine lateralization) or creation of space orthodontically for the prosthetic replacement of the missing lateral incisors. As the missing teeth in the esthetic region, it requires an interdisciplinary approach including specialists in orthodontics, prosthodontics, operative dentistry, and periodontist.

For decision-making between these two options, various factors should be considered when establishing a treatment plan. These factors include the size, shape and colour of the canine, location, age of the patient, profile of the patient, smile line, arch length tooth size discrepancy, ridge thickness, existing occlusion, patient's expectation from treatment, and cooperation during the treatment. This is to achieve a balanced dentition and optimal esthetic outcomes [9–12].

When comparing the above-mentioned treatment options, it is preferable to close the missing lateral incisor by lateralization of the canine and reshaping it to resemble the lateral incisor. This option economically costs less as well as less invasive. This will improve the function and esthetics.

## 2. Case Presentation

### 2.1. Diagnosis and Etiology

**Figure 1:**
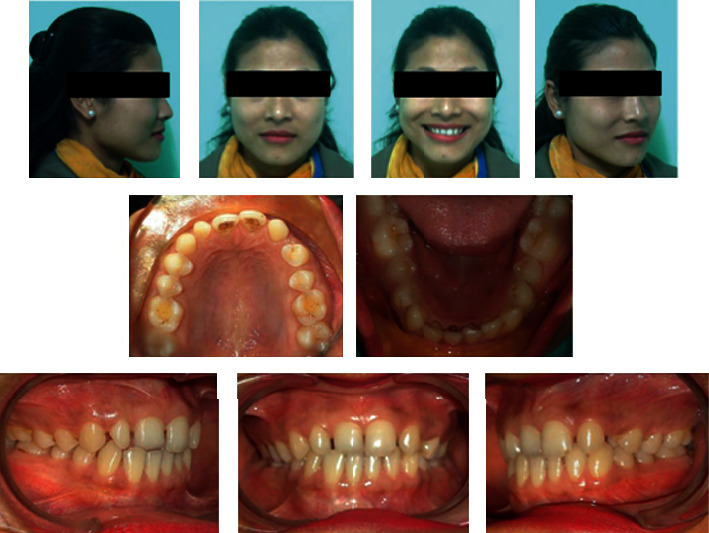
Pretreatment intraoral and extraoral photographs.

A 22-year-old female patient was referred for orthodontic consultation. Her chief complaint was the presence of a gap due to a missing tooth in the front region in the upper jaw. She had no relevant family history, no significant prenatal and postnatal history, no medical history, and no history of parafunctional habits. She was very conscious of the space present in the front teeth and her smile (Figure 1).

On clinical examination, she had a straight profile with a symmetric face and competent lips. Intraoral examination revealed class I molar relationship bilaterally, missing left maxillary lateral incisor, upper right peg lateral incisor, and gap between the teeth in the upper front region.

Both maxillary and mandibular arches were U-shaped, spacing in the maxillary arch, and the lower arch was skewed on the left buccal segment.

**Figure 2:**
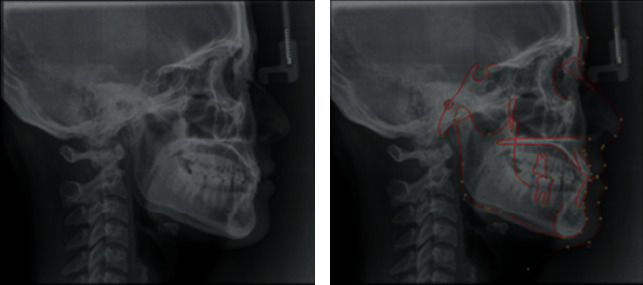
Pretreatment lateral cephalograms.

The cephalometric analysis indicates skeletal class I relation (Figure 2) with an ANB angle of 2° and nearly normal growth pattern, as shown by an FMA of 22° and SN-GoGn of 29°, nearly normally inclined and normally positioned maxillary incisor and nearly normally inclined and normally positioned mandibular incisors with obtuse nasolabial angle.

**Figure 3:**
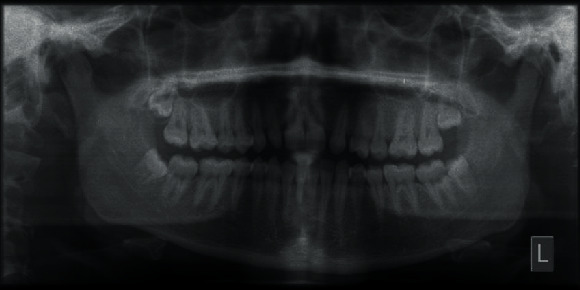
Pretreatment orthopantomogram.

The panoramic radiograph revealed a missing upper lateral incisor on the left side and the presence of all third molars. The overall alveolar bone level was within normal limits (Figure 3).

### 2.2. Treatment Objectives

The treatment objectives were to close the spaces, align irregular teeth in both jaws and replace the missing upper left lateral incisor by lateralization of 23, achieve optimal occlusion, and improve the esthetics.

### 2.3. Treatment Plan

The patient had a skeletal class I pattern and nearly normal growth pattern; hence, corrective orthodontics was planned.

Two treatment options were presented to the patient.

Option 1: creation of space for missing lateral incisor followed by a prosthesis.

Option 2: closure of missing left lateral incisor space by lateralization of canine followed by reshaping of the canine to resemble the lateral.

Both the treatment options were discussed. All the pros and cons of both the options were fully explained to the patient. The patient selected the second option.

### 2.4. Treatment Progress

Both the maxillary and mandibular teeth were banded and bonded with fully programmed preadjusted 0.022 slot MBT prescription brackets. The canine brackets on the upper left side are inversed to have +7° torque on the left upper canine which matches nearly with the torque of the upper lateral incisor tooth. Apart from this, the bracket on the upper left canine is positioned slightly gingivally to match with the gingival zenith of the contralateral lateral incisor. The first premolar bracket is positioned slightly distal to hide the palatal cusp of the first premolars on the left side and to give the cervical prominence as that of the canine.

**Figure 4:**
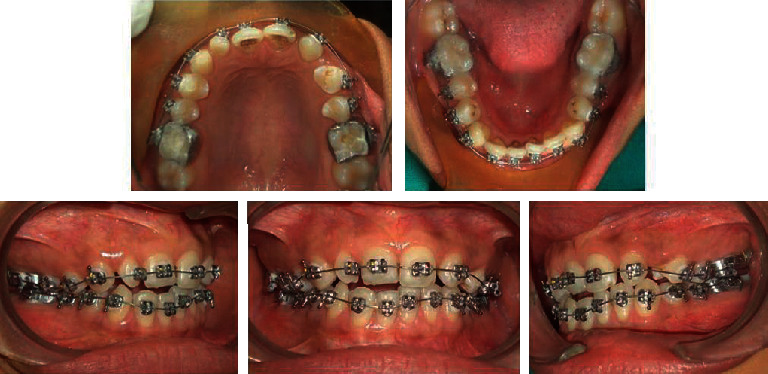
Midtreatment photographs.

**Figure 5:**
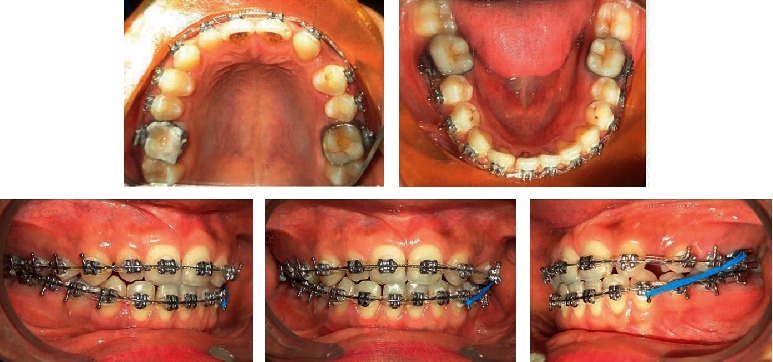
Midtreatment photographs.

**Figure 6:**
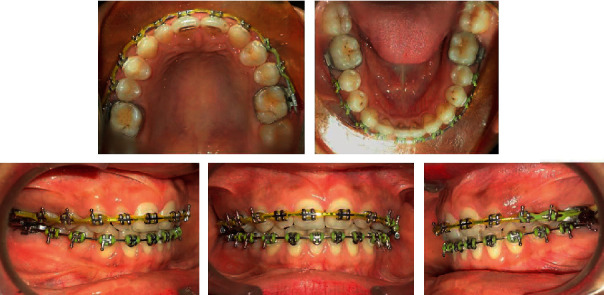
Midtreatment photographs.

The arches were aligned using the following sequence of archwires: 0.014^″^ NiTi and 0.016^″^ NiTi (Figure 4). Later, 0.018^″^ ss wire followed by 0.019 × 0.025^″^ ss wire was placed to level and express the prescription of the bracket (Figures 5 and 6). The upper left canine was protracted in the place of the lateral incisor and followed later by the first premolar using E-chain and consequently with light class III elastics (1/4^″^ 3.5 oz) for posterior segment protraction. In subsequent visits, the tip and the canine convexity were ground to simulate with the contralateral right lateral incisor and the palatal cusp tip of the upper left premolar was also ground to look like the canine tooth and to allow lateral movement. Finishing and detailing were done, and the appliance was debonded. Mild stripping of canine and premolar on the upper left side was performed to correct Bolton's discrepancy. The total treatment time was 14 months. In the retention phase, fixed bonded retainers were placed in both the arches.

### 2.5. Treatment Results

**Figure 7:**
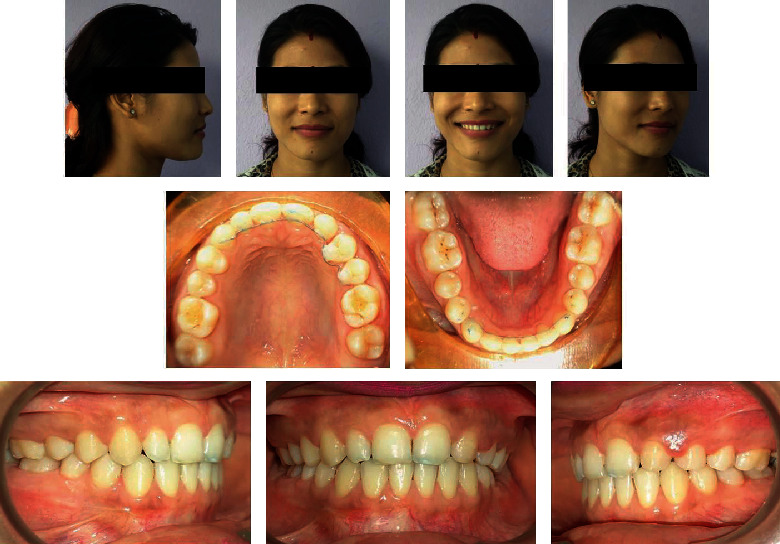
Posttreatment extraoral and intraoral photographs.

The posttreatment facial photographs exhibited a remarkable improvement of facial esthetics. The patient's smile was improved (Figure 7).

Intraorally, an optimal overbite and overjet relationship was established. A well-interdigitated buccal occlusion with class I molar relationship on the right side and class II molar relationship on the left side with class I canine relationship on both sides and coincided upper and lower dental midlines with the facial midline were achieved. There was canine guidance in lateral excursions with proper anterior guidance without balancing side interferences.

**Figure 8:**
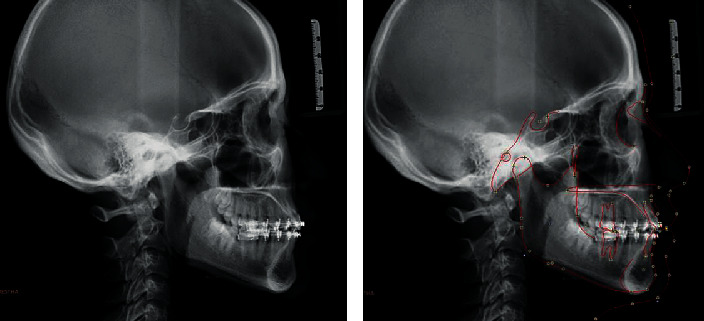
Posttreatment cephalograms.

The posttreatment cephalometric radiograph (Figure 8) showed significant changes in the dental and skeletal measurements after treatment.

**Figure 9:**
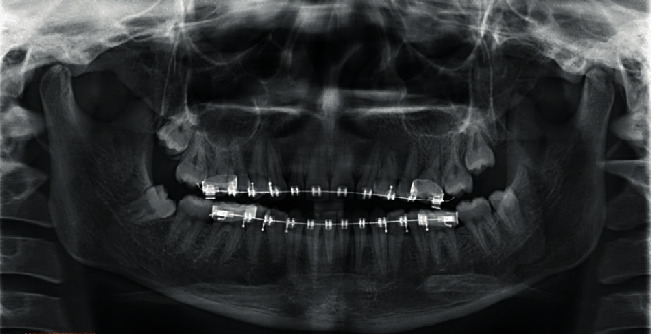
Orthopantomogram at the end of treatment.

The pretreatment and posttreatment cephalometric parameters are presented in Table 1. A panoramic radiograph was taken just before debonding to check for root parallelism (Figure 9).

## 3. Discussion

Maxillary lateral incisor agenesis is the most common developmental dental anomaly. As the missing tooth present in the esthetic region, it affects the patient's social behavior, confidence, quality of life, and professional performance; hence, management is very challenging and needs a multidisciplinary approach [13]. Thus, such patients need to be treated carefully with a multidisciplinary perspective. Optimal treatment results require patient's compliance and cooperation and good teamwork performance [14].

Lateral incisor agenesis patients with an excessive gingival display in smiling, especially young ones, should not be treated with space reopening and lateral incisor implant placement. It is inconceivable that such a technique can achieve the long-term occlusal, gingival, and periodontal results in the esthetic zone that are seen with space closure. Another important advantage of the space closure alternative is that the healthy gingival tissues and intact interdental gingival papillae will change in synchrony with the patient's own teeth over a lifetime [15].

In this patient, we have used inverted MBT canine brackets on the canine to deliver +7° degree labial crown torque that matches nearly with the torque of lateral incisor on the contralateral side. On the other hand, in the patient who requires maximum labial crown toque, +17° can be obtained by using the inverted lower second premolar bracket. The advantage of using these techniques is that prior enameloplasty is not needed as the bracket base matches the surface contour of the tooth.

Other possible variations are the use of lateral incisor bracket on the canine to provide +10° crown torque on the canine and central incisor bracket on the canine to provide +17° crown torque on the canine, but it requires prior enameloplasty on the canine to seat the bracket properly, as the central or lateral brackets have flat bases.

The appropriate time to open the space to place an implant depends on a patient's facial growth. As the face grows and the mandibular rami lengthen, the teeth must erupt to remain in occlusion. Implants cannot erupt. If an implant is placed before a patient has completed his or her facial growth, significant periodontal, occlusal (infraocclusion), and esthetic problems can be created.

The timing for implant placement after the end of growth is generally about 20 to 21 years of age for men and 16 to 17 years of age for girls [9].

Restorative procedures that may include resin-bonded FPDs, cantilevered FPDs, and conventional full-coverage FPDs can be used with success other than implants in favorable situations. Although the common cause of failure might be the debonding over time [16].

Orthodontic space closure of missing upper lateral incisor with canine lateralization can produce excellent long-term treatment results by performing the optimal torque control, differential intrusion of the first premolars and extrusion of the canines, gradual grinding of the canine cusps and buccal curvature, bleaching, minor surgical procedure for crown lengthening, and additive reshaping of the six anterior teeth using either ceramic veneers or composite [10, 15, 17–20].

Although this case requires some adjunctive procedure to improve the esthetics, the patient denied it as she is satisfied with the present achieved results.

The major advantages of orthodontic space closure in young patients with missing lateral incisor and a coexisting malocclusion are the possibility to complete treatment in early adolescence and the permanence of the finished result. Space closure treatment is considered to be less invasive, to be finished within a relatively short period of time after orthodontic therapy, and to avoid being a regular customer in the future to the dentist. The space closure with natural teeth will allow the dentition to adapt to the continuous facial changes over the patient's life [21].

Although this case report requires the patient some adjunctive procedure to improve her esthetics, she denied it as she is satisfied with the achieved results.

## 4. Conclusion

The choice of treatment option in patients having missing maxillary lateral incisor depends on various factors that need careful treatment planning with a multidisciplinary approach as the space is present in the esthetic region of the jaw. Space closure with canine lateralization option seems less costly and less invasive, treatment can be completed relatively in a short period of time, and its adaptation with the facial changes throughout life without having an artificial prosthesis.

## Figures and Tables

**Table 1:** Comparative cephalometric parameters.

Cephalometric parameters	Clinical norms	Pretreatment values	Posttreatment values
SNA	82 ± 2°	85°	85°
SNB	80 ± 2°	83°	83°
ANB	2 ± 2°	2°	2°
Wits	0-(-)1 mm	1 mm	1 mm
FMA	25 ± 2°	22°	23°
SN-GoGn	32 ± 2°	29°	30°
Max.I-NA	22 ± 2°	23°	22°
Man.I-NB	25 ± 2°	21°	22°
LI-A-Pog	2.7 ± 1.7mm	1 mm	1 mm
IMPA	90 ± 2°	87°	90°
Interincisal angle	134°	133°	132°

## Data Availability

The datasets used and/or analyzed during the current study are available from the corresponding author on reasonable request.
